# Ethics competences in the undergraduate medical education curriculum: the Spanish experience

**DOI:** 10.3325/cmj.2016.57.493

**Published:** 2016-10

**Authors:** Guillermo Ferreira-Padilla, Teresa Ferrández-Antón, Fernando Lolas-Stepke, Rut Almeida-Cabrera, Joan Brunet, Joaquim Bosch-Barrera

**Affiliations:** 1 Faculty of Health Sciences, Research and innovation in health care and resources, University of Alicante, Alicante, Spain; 2 Närhälsan Primary Care Center, Skövde, Sweden; 3 Pediatric Clinic, Skaraborgs Sjukhus Skövde, Skövde, Sweden; 4 Interdisciplinary Center for Bioethics and Psychiatric Clinic, University of Chile, Santiago de Chile, Chile; 5 Department of Medical Oncology, Catalan Institute of Oncology, Doctor Josep Trueta University Hospital, Girona, Spain; 6 Girona Biomedical Research Institute (IDIBGi), Girona, Spain; 7 Department of Medical Sciences, Medical School, University of Girona, Girona, Spain

## Abstract

**Aim:**

To investigate if there are differences in medical ethics education between different schools of medicine in Spain, specifically between private and public schools and between recently founded schools and older ones.

**Method:**

The curricula of medical degrees from all Spanish faculties were reviewed for the 2014/2015 academic year, identifying subjects concerning bioethics, deontology, and ethics. We identified the type of teaching, format and method of the course, the number of credits and hours, and the school year of each subject. An analysis with descriptive parameters and the Cohen’s coefficient (*d*) was performed.

**Results:**

All medical schools in Spain (n = 44) were included. A mean of 3.64 European Credit Transfer and Accumulation System (ECTS) credits was specifically devoted to ethical values teaching in Spain. Private medical schools offered more credits than public ones (6.51 ECTS vs 2.88 ECTS, relevant difference: *d* = 2.06>>0.8), and the 10 most recently founded medical schools offered more credits than the 10 oldest (5.86 ECTS vs 2.63 ECTS, relevant difference: *d* = 1.43 > 0.8). A mean of 36.75 hours was dedicated to ethics education.

**Conclusions:**

Although ethics education is incorporated into the training of future Spanish physicians, there is still notable heterogeneity between different medical schools in the time devoted to this topic.

Service professions demand from their practitioners knowledge and the practice of diverse types of values (1). Their practitioners must be familiar with and respect values intrinsic to the human condition, such as dignity of the person, acceptance of autonomy, the intention to do well and avoid damage, and justice (2).

The exercise of medicine has clear ethical implications, and physicians need conceptual tools to analyze and resolve the ethical dilemmas encountered in their clinical practice (3). A key component of professional behavior is the physician’s ability and willingness to act in accordance with accepted moral norms and values. Therefore, medical students must receive ethical and/or bioethical education during their medical training at medical school (4).

In recent decades, a complex set of notions has been incorporated to educational practices under the term bioethics. Widening and deepening traditional professional deontology, these notions strive at fostering knowledge of values and the humanization of the professions, especially those related to health care (3,4). While adequate knowledge of norms, regulations, and principles is essential, it is not enough (5).

Although the relationship between medicine and ethics has existed since ancient times, as highlighted by the Hippocratic Oath (460-370 BC) (6), ethics education was not a formal requisite of medical schools until a few decades ago. In 1985, the DeCamp Report argued that basic instruction in medical ethics should be a requirement in all US medical schools (7). Currently, the major accrediting bodies for US medical schools and residency programs state that medical education programs must include instruction in medical ethics (8,9). Similarly, in 1999, the World Medical Associations assembly recommended that all medical schools should include the teaching of medical ethics and human rights as an obligatory course in their curricula (10). Few empirical studies address the degree to which ethics education has been incorporated into the formal curricula of medical schools. In 2000, only 78% of US and Canadian medical schools incorporated ethics into required preclinical courses (11). Similar data were obtained in Europe in 2007, with 84% of medical schools having at least one ethics module (12).

Due to the heterogeneity of approaches to medical education and the scarce information available, in this article we assess the state of medical ethics education in Spain after the university adopted the European Higher Education Area plan in 2010 (“Bologna process”) (13). This is the first report on the medical ethics education situation in Spain. In our study, we considered specific courses addressing bioethics, deontology, and ethical medicine (BDE) as subjects related to ethical values. Our initial hypothesis was that medical ethics education would be heterogeneous between different schools of medicine in Spain. Also, we wanted to analyze if there were differences between private and public schools of medicine or between recently founded and older schools.

## Methods

2569 subjects belonging to 44 medical degree curricula from accredited Spanish universities were analyzed. We used the same methodology that was used in a similar study about communication skills teaching in the Spanish medical schools (14).

### Design

This observational, descriptive-comparative, and transverse study was conducted in the teaching university environment of undergraduate medical education in Spain. The entire population of medical schools was considered. Two official databases were consulted: the Ministry of Education, Culture and Sports, Government of Spain and the National Conference of Deans (15,16). The study population included all Spanish medical schools that met the following inclusion criteria:

1) that they taught the official Degree in Medicine, as approved by the National Agency for Quality Assessment and Accreditation of Spain (ANECA), during the 2014-2015 school year.

2) that they offered the needed information (curricula and teaching guides) through the websites of each university and/or via e-mail or telephone.

Exclusion criteria were as follows:

1) the pertinent information about the variables of study was unobtainable using the previously mentioned methods.

2) the medical schools were under construction and/or accreditation by the ANECA.

3) the university did not provide undergraduate medical education during the 2014-2015 school year, despite the favorable approval by the ANECA.

4) the ethics subjects were integrated into non-Bologna Medical Degrees (in extinction).

It was not necessary to calculate the size of the sample because representative sample was not analyzed, but the whole population of Spanish medical schools (100%). Studies of statistical inference with the application of hypothesis tests were also not necessary to extend the sample information to the supposed total population with a confidence level of 1-α. Additionally, by evaluating the whole population, the random error was zero. In summary, because it was possible to know and work with the entire study population, inferential statistics became unnecessary.

### Collection of the information

With the aim of contrasting and confirming the data, two of the authors performed a systematic and independent study spanning two different time periods (October 2014 and June 2015). This part was developed in three stages:

*Stage 1.* The free-access databases of the Spanish Ministry of Education and the National Conference of Medical Schools’ Deans (15,16) were consulted with the aim of determining the number of existing medical schools during the 2014-2015 school year.

*Stage 2.* The curricula and teaching guides were exhaustively reviewed by accessing the websites of all medical schools. Instructors of the subjects at the University of Salamanca and CEU San Pablo University were contacted by e-mail and telephone to obtain the necessary information (in this case there was no public teaching guide but only the information in the curricula was obtained). Contact by e-mail was successfully established with the University of Salamanca. Within the curriculum structure, the research focused on the contents about BDE, regardless of the name of the subject. At this point, of the total analyzed subjects (2569), 2513 (97.82%) were excluded for including other contents. By agreement, a checklist was created to define how to consider each subject in relation to the variables, and this checklist was meticulously followed by each of the two authors in charge of this stage (Supplementary material 1)(web extra material 1).

*Stage 3*. A thorough study about the White Book: Degree in Medicine was conducted (17), paying special attention to the content related to BDE. This book is a comprehensive report by the National Conference of Deans of Medicine of Spain in which a degree-design is elaborated according to the European recommendations. Among other things, the number of credits and the percentage of various generic blocks and the corresponding number of hours (according to the European Higher Education Area) is proposed. This book describes the competences that a medical student must acquire during the undergraduate medical education.

## Study variables

### Qualitative variables

1) Presence of subjects about BDE. By “presence” we considered the existence of this education in any of its ways (see point 2 “type of teaching”). In some cases, there was only a single chapter/lecture on BDE in some subjects. It was determined that a minimum value of credits had to be included as a lower limit for considering whether the university taught BDE (this affected only the combined teaching). This lower limit was defined as at least 1.5% of the total subjects’ credits. Thus, credits for this teaching as a transverse competence were excluded.

2) Type of teaching: “exclusive” or “combined.” This variable is equivalent to “specific competence” and “transverse competence,” respectively. “Exclusive teaching” was defined as teaching only contents about BDE (ie, “Bioethics” or “Professionalism in Medicine”) within a subject. “Combined teaching” was defined as teaching BDE along with other types of contents within a subject (ie,“Ethics and Law in Medicine” or “Ethics and Communication Skills”).

3) Teaching methodology: “theoretical” or “mixed” teaching. “Theoretical teaching” included only lectures (without practical classes), while “mixed teaching” included lectures and practical classes. The number and the quality of practical classes were considered by carefully analyzing the methods used to teach and evaluate these classes.

4) Type of subject: “core education,” “compulsory,” or “optional.”

“Core education.” Courses that teach the basic aspects of a branch of knowledge. According to the Spanish system, each school degree must contain at least 60 credits of basic training (core), 36 of them linked to the branch of knowledge to which the degree belongs. Example: “Physiology,” “Anatomy,” or “Statistics Applied to Health Sciences.”

“Compulsory.” Selection of specific medical subjects that all students are required to complete before they can move on to the next level in the medical degree. Example: “Dermatology,” “Gynecology and Obstetrics,” or “Ophthalmology.”

“Optional.” The students choose freely from the entire catalog of subjects offered by each university.

5) Duration of the subject: “annual,” “four months long,” or “three months long.”

6) School year: 1 to 6.

### Quantitative variables (continuous)

1) Number of credits. Only those credits linked to specific competences (discarding the transverse ones) were considered. These credits are based on the European Credit Transfer and Accumulation System (ECTS), based on the European Higher Education Area. The total national mean of BDE credits was compared to the 7.0 credits recommended by ANECA for the “professional and ethical values” part. We obtained the percentage of credits reserved solely for BDE among these 7.0 recommended credits.

2) Number of hours. Only the classroom hours of the subject were analyzed. Calculations were performed according to the correspondence between ECTS and hours established by each medical school. In those schools where the specific number of hours dedicated to BDE teaching was not detailed and the correspondence between credits and hours was not described, the mean of 27.5 hours (25-30 hours) and 35% of presence (30%-40%) were used, following the European Higher Education Area’s recommendations.

### Data analysis

The data summary was created using Microsoft® Excel® 2010 (Microsoft Corporation, Redmond, WA, USA). The statistical package SPSS® version 19.0 (IBM SPSS, Inc., Chicago, IL, USA) was used to analyze the data. First, a wide descriptive statistical study was performed in which the following parameters were analyzed: median with interquartile range, mean with standard deviation, mode, and the Fisher asymmetry coefficient. The Cohen’s coefficient (*d*), which is the most used parameter in studies about the science of education to calculate the size of the effect (18), was applied to calculate the magnitude of the differences between distinct parameters. This calculation was especially useful for determining the size of the effect in the categorized differences. To synthesize the data, the analysis strategy was based on identifying which medical schools presented subjects about BDE in their curricular structure. The study was further extended with the collection of information about teaching characteristics, such as the number of credits, the number of hours, the type of education and subject, the methods applied to teach the practical education, or the placement of the course within the curriculum. These results were compared with other published reports and articles of similar content (12,17,19,20).

## Results

There were 44 medical schools in Spain, 10 private (22.73%) and 34 public/state (77.27%) ones. 7 (15.91%) of these medical schools officially recognized religious influence and the remaining 37 (84.09%) were secular schools. International University of Catalonia is a private university that, although it is not an officially recognized religious university, is inspired by Christian humanism and offers a Catholic Chaplaincy service (Table 1).

**Table 1:** Bioethics, deontology, and ethics teaching in Spanish medical schools

General description	n	%
**Total number of medical schools in Spain**	44	100
**Ownership of medical schools**		
private	10	22.7
public	34	77.3
**Does this medical school teach bioethics, deontology, and ethics?**		
yes	44	100
no	0	0
**Description of teaching in bioethics, deontology, and ethics**		
**Credits by Medical Schools (n = 43)**		
0-0.99	3	7
1-1.99	3	7
2-2.99	8	18.6
3- 3.99	17	39.5
4-4.99	3	7
5-5.99	2	4.7
6-6.99	4	9.3
≥7.0	3	7
**Analyzed subjects total**	2569	
**Subjects about bioethics, deontology, and ethics**	56	2.2
Type of teaching		
exclusive	27	48.2
combined	29	51.8
Type of subject		
core education	11	19.6
compulsory	44	78.6
optional	1	1.8
Duration		
annual	3	5.4
four months long	51	91.1
three months long	2	3.5
School year		
first	6	10.7
second	20	35.7
third	15	26.8
fourth	9	16.1
fifth	4	7.1
sixth	2	3.6
		

This study included all medical schools. However, we were unable to obtain information on the methodology and the number of credits and hours from CEU San Pablo University (private and religious). The only document that was published on its website was the curricula, not the teaching guide (where all the information about our variables appeared).

### Qualitative variables

*The presence of subjects about BDE*. Of 2569 analyzed subjects, 56 (2.18%) were dedicated to this type of education. BDE were taught in 100% (44/44) of the medical schools. In all schools, there was at least 1 subject about these topics, in 12 (22.27%) schools there were 2 subjects, while in no schools there were more than 2 subjects (Table 1).

*“Exclusive” or “combined” teaching type*. “Combined teaching” was employed in 29 (51.79%) subjects, while “exclusive teaching” was employed in 27 (48.41%). The subject in which “combined teaching” was most frequently employed was Legal Medicine and Healthcare Communication (with 9 and 6 subjects, respectively). 20 (45.89%) medical schools employed *“*exclusive teaching” and 21 (47.73%) employed “combined teaching.” The remaining 3 (7.14%) medical schools simultaneously employed both types of teaching (Table 1).

*Teaching methodology: “Theoretical” or “mixed.”* “Mixed” methodology was the most used type, in 32 (74.42%) schools. 5 (11.63%) used only “theoretical” methodology and 6 (13.95%) integrated both methodologies. All the private medical schools used ***“***mixed***”*** methodology, and 3 of them also used “theoretical” teaching (Table 2). The most common and frequently used teaching tools were seminars and the discussion-resolution of cases (ie, ethical issues in clinical practice).

**Table 2:** Teaching and evaluation methods of the practical content of bioethics, deontology, and ethics

General description	n
Seminars	22
Discussion and resolution of clinical cases	19
Videoforums	9
Problem-based learning	3
Critical readings	3
Portfolio on practical content	2
Simulating the operation of an Ethics Committee	2
Conferences on bioethics	2
“Lectures by experts”	1
Online course on bioethics and ethics	1
Clinical practice in hospital	1
Role-playing	1
Other unspecified activities	10

Practical test	n
Medical schools with a practical test (Group 1)	9
Medical schools without a practical test (Group 2 and 3)	34
Assessment of practical content without a practical test	18
Without examination and no evaluation	16
**Methods used in the practical evaluation**	**n**
Discussion and resolution of clinical cases	6
Problem-based learning	2
Objective Structured Clinical Examination	1

There were three different groups regarding the practical content of the course:

▪ Group 1: With examination. 9 (20.93%) medical schools integrated a practical test about practical content. Just 1 school performed an Objective and Structured Clinical Evaluation. 2 (4.65%) examined their students through problem-based learning.

▪ Group 2: Assessment of practical content without a practical exam. 16 (37.21%) medical schools considered the students’ work during their internship when evaluating them. The students’ attendance, active participation, and the portfolio or other kind of work that must be delivered at the end of the training were considered.

▪ Group 3: No examination and no evaluation. 18 (41.86%) medical schools neither had a test nor considered the work of the students during their internship. Two universities used a practical test only for the students who had not undertaken internships (Table 1).

*Type of subject: “Core education,” “compulsory,” or “optional.”* The *“*compulsory” format was the most commonly used (n = 32; 72.72%), followed by “core education” (n = 9; 20.46%). Only 3 (6.81%) universities employed different formats for the 2 subjects on BDE that they offered. Only 1 university employed the “optional” format (Table 1).

*Duration of the subject: “Annual,” “four months long,” or “three months long.”* The majority of schools (n = 39; 88.64%) had “four months long” subjects, 3 had annual subjects, and 2 had “three-month-long” subjects (Table 1).

*School year: 1 to 6.* Most of the subjects (n = 41; 73.21%) with contents about BDE were taught during the first cycle (first to third school year) (n = 27; 61.36%), with the second year representing the mode (n = 20; 35.71%). Only 2 (3.57%) courses were taught in the last school year (Table 1). Among the 12 (27.27%) medical schools that had 2 subjects on BDE, the most used combination was the third and fourth school years.

### Quantitative variables

**Figure 1:**
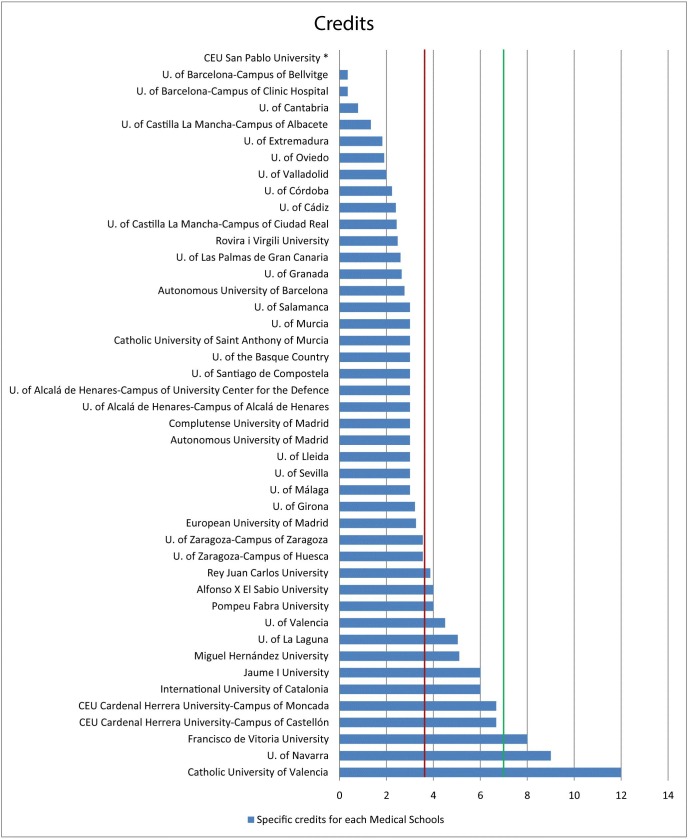
Total European Credit Transfer and Accumulation System (ECTS) credits for each medical school. The green line indicates the 7.0 credits recommended by National Agency for Quality Assessment and Accreditation of Spain that should be devoted to “professional and ethical values.” The red line indicates the mean of number of ECTS credits devoted to teaching on bioethics, deontology, and ethics at medical schools in Spain. *Information from CEU San Pablo University could not be obtained for this parameter. Medical schools with officially recognized religious influence: Catholic University of Valencia, San Antonio Catholic University of Murcia, CEU-Cardinal Herrera University-Campus of Castellón, CEU-Cardinal Herrera-Campus of Moncada, CEU San Pablo University, Francisco de Vitoria University, and University of Navarra.

*Number of credits*. The mean number of credits was 3.64 ECTS (standard deviation [SD] = 2.24), with a wide range (0.35-11.65 ECTS credits) (Figure 1). This number represents a mean of 1% of the total of 360 ECTS credits that comprise the degree of medicine. The mean number of credits in private medical schools was 6.51 ECTS (SD = 2.92) and the mean number of credits in public/state schools was 2.88 ECTS (SD = 1.22). Only 3 universities exceeded the 7.0 credits recommended by ANECA for the teaching of BDE (17).

Regarding the type of institution, the 6 officially recognized religious medical schools had a mean of 7.56 ECTS (SD = 2.98), while the 37 secular institutions had a mean of 3.00 ECTS (SD = 1.29). The 10 most recently founded universities had a mean of 5.86 ECTS credits (SD = 2.72), while the 10 oldest ones had a mean of 2.63 (SD = 1.36).

The Fisher coefficient of asymmetry (γ = 1.72 > 0) showed a distribution with right asymmetry. Thus, more medical schools offered fewer than the mean (3.64 credits) number of credits. Specifically, in 50% of Spanish medical schools, the number was equal to or fewer than 3.0 ECTS credits.

**Figure 2:**
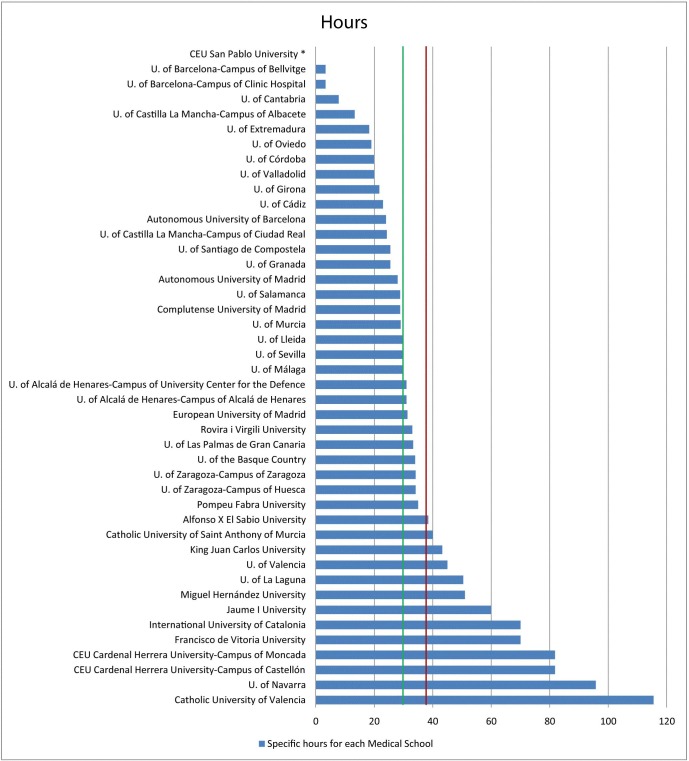
The total number of hours for each medical school. The green line indicates the 30-hour threshold recommended by UNESCO. The red line indicates the mean of number of hours devoted to teaching on bioethics, deontology, and ethics at medical schools in Spain. *Information from CEU San Pablo University could not be obtained for this parameter. Medical schools with officially recognized religious influence: Catholic University of Valencia, San Antonio Catholic University of Murcia, CEU-Cardinal Herrera University-Campus of Castellón, CEU-Cardinal Herrera-Campus of Moncada, CEU San Pablo University, Francisco de Vitoria University, and University of Navarra.

*Number of hours*. The mean of number of hours was 36.75 (SD = 23.19), with a wide range (3.34-115.5 hours) (Figure 2). The mean number of hours in private medical schools was 67.89 (SD = 28.45) and the mean number of hours in public/state ones was 28.50 (SD = 12.39). 6 religious medical schools had 80.58 (SD = 25.33) hours and 37 secular ones had 29.64 (SD = 12.89) hours. In addition, the 10 most recently founded universities had a mean of 60.96 (SD = 26.06) hours, while the 10 oldest ones had 25.32 (SD = 13.27) hours.

### Comparisons using the Cohen’s coefficient

Cohen’s coefficient was calculated to determine the size of the effect of the differences (Table 3). There were positive relevant differences in favor of private, religious and most recently founded medical schools. Private medical schools offered more credits than public ones (6.51 ECTS vs 2.88 ECTS, *d* = 2.059>>0.8). Religious medical schools offered more credits than secular ones (7.56 ECTS vs 3.0 ECTS, respectively, *d* = 2.755>>0.8) and the 10 most recently founded medical schools offered more credits than the 10 oldest ones (5.86 ECTS vs 2.63 ECTS, *d* = 1.430 > 0.8).

**Table 3:** Significance of differences: Cohen’s coefficients*

Difference in the mean with SD of BDE credits between private and public medical schools	Result	Difference
Mean of credits at private MS (6.51; SD = 2.92) vs mean of credits at public MS (2.88; SD = 1.22)	*d* = 2.059>>0.8	Relevant
Mean of credits at private MS (6.51; SD = 2.92) vs total MS’ mean of credits (3.64; SD = 2.24)	*d* = 1.186 > 0.8	Relevant
Mean of credits at public MS (2.88; SD = 1.22) vs total MS’ mean of credits (3.64; SD = 2.24)	*d* = 0.403 < 0.5	Small

Difference in the mean with SD of BDE credits between religious or secular schools	Result	Difference
Mean of credits at religious MS (7.56; SD = 2.98) vs mean of credits at secular MS (3.00; SD = 1.29)	*d* = 2.755>>0.8	Relevant

Difference in the mean with SD of BDE credits between the 10 oldest MS and 10 most recently founded schools	Result	Difference
Mean of credits at the 10 oldest MS (2.63; SD = 1.36) vs mean of credits at the 10 most recently founded schools (5.86; SD = 2.72)	*d* = 1.430 > 0.8	Relevant

Difference between the mean with SD of credits and the number of credits recommended by ANECA	Result	Difference
Total MS’ mean of credits (3.64; SD = 2.24) vs ANECA’s recommended credits (7.0)	*d* = 2.096>>0.8	Relevant
Mean of credits at private MS (6.51; SD = 2.98) vs ANECA’s recommended credits (7.0)	*d* = 0.395 < 0.5	Small
Mean of credits at public MS (2.88; SD = 1.22) vs ANECA’s recommended credits (7.0)	*d* = 5.015>>0.8	Relevant

Difference between the mean with SD of hours and the number of hours recommended by UNESCO	Result	Difference
Total MS’ mean of hours (36.75; SD = 23.19) vs UNESCO’s recommended hours (30.0)	*d* = 0.359 < 0.5	Small
Mean of hours at private MS (67.89; SD = 28.45) vs UNESCO’s recommended hours (30.0)	*d* = 2.415>>0.8	Relevant
Mean of hours at public MS (28.50; SD = 12.39) vs UNESCO’s recommended hours (30.0)	*d* = 0.179 < 0.2	Small

*ANECA – National Agency for Quality Assessment and Accreditation of Spain; BDE – teaching on bioethics, deontology, and ethics; MS – medical school; UNESCO – United Nations Educational, Scientific and Cultural Organization; SD – standard deviation.

†Cohen’s coefficient (*d*) interpretation: *d*<0.2 = small difference (small size effect); *d*>0.5 = moderate difference (medium size effect); *d*>0.8 = relevant difference (high size effect).

The total national mean of credits and the mean regarding the type of institution (private or public/state) were lower than the number proposed by ANECA (7.0 ECTS) (17): *d* = 0.395 < 0.5 for private medical schools and *d* = 5.015>>0.8 for public medical schools.

The total national mean number of hours (*d* = 0.359 < 0.5) and the mean number in the private medical schools *(d* = 2.415>>0.8) was higher than the 30 hours recommended by UNESCO (19). Cohen’s coefficient was calculated to determine the size of the effect of the difference between the mean number of hours in Spain and the number recommended by UNESCO. A positive difference in favor of the total mean hours for private medical schools was observed (*d* = 2.415>>0.8) (Table 3). Additionally, all the private medical schools (n = 9) were among the 22 (51.16%) institutions that had more hours than recommended by UNESCO; the 6 medical schools that taught the most hours were private universities, with 5 of them being religious.

## Discussion

Our study found that there were differences in ethics education between medical schools in Spain.

ANECA, the official organization that certifies the teaching of medicine at medical schools in Spain, has established that 7.0 credits should be devoted to “professional and ethical values” in the medical degree curricula. These 7.0 credits include not only ethical medicine or bioethics, but also legal medicine. ANECA has not defined the exact proportion of credits dedicated to each of these contents, which contributes to the heterogeneity among the medical schools observed by our study. Our study showed that approximately half (51.8%) of the subjects devoted to ethical values were mixed with legal medicine content. The mean number of credits specifically devoted to ethical values teaching in Spain was 3.64 ECTS, which represent a mean of 1% of the total credits that comprise the degree of medicine. Significantly more credits (6.51 ECTS) were offered in private than in public (2.88 ECTS) medical schools. In Spain, most of the private universities have religious designation (Christian Catholic religion), which may explain why ethics and morality are more represented in their curricula. Furthermore, religious medical schools dedicated a mean of 7.56 ECTS to BDE compared with a mean of 3.00 ECTS in secular ones.

In 2008, the UNESCO Bioethics Core Curriculum defined that there should be a minimum of 30 hours (in terms of teaching hours and contents) dedicated to appropriate bioethics teaching for medical university students (19). This endpoint is achieved throughout Spain, with a mean of 36.75 hours, and this value is doubled when we consider only private medical schools (67.89 hours). Unfortunately, 41% of the medical schools are still below this indicator. However, we only considered the classroom hours dedicated to the subject, which can underestimate the real number of hours devoted to BDE, for example in universities that use problem-based learning methodologies.

The first exploratory report on ethics teaching in Europe in 2007 found that the mean time invested in ethics teaching was 44 hours during the overall curriculum, and that 84% of medical schools had at least one ethics module (12). More recently, a study of medical schools curricula in southeast Europe reported a mean of 27.1 teaching hours of medical ethics and bioethics, with the national means ranging from 47.5 hours in Croatia to 14.8 hours in Serbia (20). This study analyzed data from few medical schools for each country (for example 2 of the 4 medical schools of Croatia), which limited the study of variability within the same country. There is no doubt that Croatia is very much involved in integrating BDE teaching in their medical schools and interested in health care communication (21,22).

We observed that the number of credits devoted to teaching ethical values in the most recently founded medical schools was nearly two times higher than in older schools. This result could be an indicator of the progressive recognition of the importance of ethics instruction in medical schools. Another explanation could be the difficulties of assigning ECTS credits to new subjects devoted to BDE in older medical schools. In older universities, a fixed number of total credits is given to students during their studies. If there is an increase in the number of ECTS credits in favor of BDE subjects, then it could only be at the expense of other subjects such as “Law Medicine” or “Physiology.” This is not acceptable at the moment, because re-assigning these credits could decrease the number of planned hours for these subjects and lead to teacher layoffs.

There is no single, best pedagogical approach for teaching medical ethics and professionalism, so teaching methods need to be flexible and varied (4). In Spain different educational strategies are used, with seminars, discussion and resolution of clinical cases, and videoforums being the most used techniques. Very few medical schools use problem-based learning and role-play scenarios, which can help students translate their medical knowledge into skills before they encounter the actual patients (23). Additionally, only one-fifth of medical schools are performing a practical evaluation of this subject. It is crucial that ethics and professionalism education move learners from knowledge acquisition and skills development to behavior changes, with excellent patient care as the primary goal. Practical evaluation could help meet this endpoint (4).

Nevertheless, one or two subjects on BDE during the undergraduate medical education are not enough. All disciplines should be taught in a bioethical context. In all areas, the ethics of scientific communication must play an important role in teaching (24).

Some of the limitations of our study were: 1) the absence of previous studies to compare our results with other experiences or with the evolution of the teaching of BDE in Spain; 2) limited information available from some schools of medicine; and 3) possible interview bias during the collection of the data. Although the focus in this article was on medical ethics education during medical school, we acknowledge that ethics and professionalism education are not a one-time, isolated event. Rather, they are an issue that requires continuing education (25). Medical ethics and professionalism should also be prioritized during residency training and reinforced post-residency through continuing medical education (4,26).

Acknowledgments We thank Dr. Prof. José M. Peinado Herreros, Dean of the Faculty of Medicine of the University of Granada and Coordinator of the ANECA White Book, for his comments and explanations that enriched this paper.

## References

[1] ABIM Foundation (2002). American Board of Internal Medicine; ACP-ASIM Foundation. American College of Physicians-American Society of Internal Medicine; European Federation of Internal Medicine. Medical professionalism in the new millennium: a physician charter.. Ann Intern Med.

[2] Beauchamp TL, Childress JF. Principles of biomedical ethics. Oxford: Oxford University Press; 2012.

[3] Carrese JA, Malek J, Watson K, Lehmann LS, Green MJ, McCullough LB (2015). The essential role of medical ethics education in achieving professionalism: the Romanell Report.. Acad Med.

[4] Doukas DJ, Kirch DG, Brigham TP, Barzansky BM, Wear S, Carrese JA (2015). Transforming educational accountability in medical ethics and humanities education toward professionalism.. Acad Med.

[5] Lolas F. Bioethics. Moral dialogue in life sciences. Santiago de Chile: Chile Editorial Universitaria; 1999.

[6] Emery AEH (2013). Hippocrates and the oath.. J Med Biogr.

[7] Culver CM, Clouser KD, Gert B, Brody H, Fletcher J, Jonsen A (1985). Basic curricular goals in medical ethics.. N Engl J Med.

[8] Liaison Committee on Medical Education. Functions and Structure of a Medical School: Standards for accreditation of medical education programs leading to the M.D. degree 2013. Available from: https://www.lcme.org/publications/functions2013june.pdf. Accessed: November 2015.

[9] Accreditation Council for Graduate Medical Education. ACGME Common Program Requirements 2013. Available from: https://www.acgme.org/acgmeweb/Portals/0/PFAssets/ProgramRequirements/CPRs2013.pdf. Accessed: November 2015.

[10] World Medical Association. Resolution on the inclusion of medical ethics and human rights in the curriculum of medical Schools world-wide 1999, Tel Aviv, Israel. Available from: http://www.wma.net/en/30publications/10policies/e8/ Accessed: November 2015.

[11] Lehmann LS, Kasoff WS, Koch P, Federman DD (2004). A survey of medical ethics education at U.S. and Canadian medical schools.. Acad Med.

[12] Claudot F, Alla F, Ducrocq X, Coudane H (2007). Teaching ethics in Europe.. J Med Ethics.

[13] Lobato RD, Lagares A, Alén JF, Alday R (2010). Implementation of the Bologna system in medical education. Current status and future prospects. Neurocirugia (Astur).

[14] Ferreira Padilla G, Ferrández Antón T, Baleriola Júlvez J, Almeida Cabrera R (2015). Communication skills in the curriculum of Medical students from Spain (1990-2014): From the Primary Health Care to the Bologna Plan. A descriptive study. Aten Primaria.

[15] Conferencia Nacional de Decanos de Facultades de Medicina. Available from: http://www.cndmedicina.com/. Accessed: November 2015.

[16] Ministry of Education. Culture and Sports of Spain. QUEDU-Degree search. Available from: https://www.educacion.gob.es/notasdecorte/busquedaSimple.action*.* Accessed: November 2015.

[17] Agencia Nacional de Evaluacion de la Calidad y Acreditación (ANECA). Libro Blanco: Título de Grado en Medicina. Peinado Herreros J, editor. Madrid; 2005. 1-596: Available from: http://www.aneca.es/var/media/150312/libroblanco_medicina_def.pdf. Accessed: November 2015.

[18] McMillan JH, Foley J (2011). Reporting and discussing effect size: still the road less traveled?. Pract Assess, Res Eval.

[19] United Nations Educational, Scientific and Cultural Organisation (UNESCO). Bioethics Core Curriculum. Section 1: Syllabus ethics education programme 2008. Available from: http://unesdoc.unesco.org/images/0016/001636/163613e.pdf. Accessed: November 2015.

[20] Mijaljica G (2014). Medical ethics, bioethics and research ethics education perspectives in South East Europe in graduate medical education.. Sci Eng Ethics.

[21] Pelčić G (2013). Bioethics and medicine.. Croat Med J.

[22] Ferreira-Padilla G, Ferrández-Antón T, Baleriola-Júlvez J, Braš M, Đorđević V (2015). Communication skills in medicine: where do we come from and where are we going?. Croat Med J.

[23] Bosch-Barrera J, Briceńo García HC, Capella D, De Castro Vila C, Farrés R, Quintanas A (2015). Teaching Bioethics to Students of Medicine with Problem-Based Learning (PBL). Cuad Bioet.

[24] Anderson MA, Giordano J (2013). Aequilibrium prudentis: on the necessity for ethics and policy studies in the scientific and technological education of medical professionals.. BMC Med Educ.

[25] Busche K, Burak KW, Veale P, Coderre S, McLaughlin K (2016). Making progress in the ethical treatment of medical trainees.. Adv Health Sci Educ Theory Pract.

[26] Wasson K, Anderson E, Hagstrom E, McCarthy M, Parsi K, Kuczewski M (2016). What ethical issues really arise in practice at an academic medical center? A quantitative and qualitative analysis of clinical ethics consultations from 2008 to 2013.. HEC Forum.

