# Supplementary material for: Ethics competences in the undergraduate medical education curriculum: the Spanish experience
Source: Croat Med J. 2016 Oct;57(5):493–503. doi: 10.3325/cmj.2016.57.493 (PMC5141466; doi:10.3325/cmj.2016.57.493)
Supplement: Supplementary Table 1 [file CroatMedJ_57_s003.pdf]

***Supplementary material* .- CHECK LIST**

- **Name of the University:**
- **Type of University:**
    - PUBLIC/STATE / PRIVATE
    - RELIGIOUS UNIVERSITY / SECULAR
- **Number of Universities in alphabetical order:**
- **Name and contact information (email) of the responsible professor:**
    - **Name:**
    - **Email:**

Qualitative variables – CHECK LIST (mark the desired answer)

1. Presence of subjects about bioethics/deontology/ethics? YES / NO
2. Type of teaching? EXCLUSIVE / COMBINED
3. Teaching methodology? THEORETICAL TEACHING / MIXED TEACHING
4. Type of subject? CORE EDUCATION / COMPULSORY / OPTIONAL
5. Duration of the subject? ANNUAL / FOUR MONTHS LONG / THREE MONTHS LONG
6. School year? FIRST / SECOND / THIRD / FOURTH / FIFTH / SIXTH

Quantitative variables – CHECK LIST (calculate separately and provide the explanation)

7. Number of credits:
8. Number of hours:

Comments:
